# Supplementary material for: Splice-Junction-Based Mapping of Alternative Isoforms in the Human Proteome
Source: Cell Rep. Author manuscript; Available in PMC 2020 Jan 15. (PMC6961840; doi:10.1016/j.celrep.2019.11.026)
Supplement: 2 [file NIHMS1546469-supplement-2.zip › DF1/PXD010154/Liver/Liver_34_RFWD2_QLEAQLEQIQK.pdf]

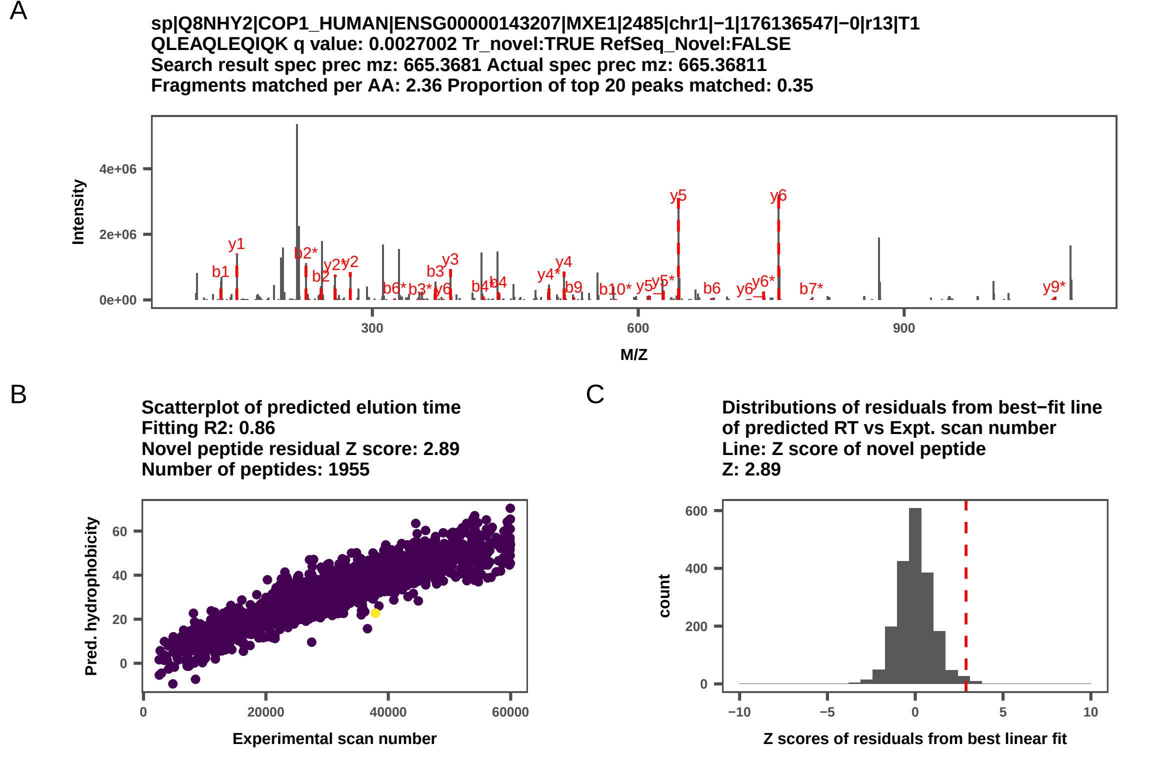
A
sp|Q8NHY2|COP1_HUMAN|ENSG00000143207|MXE1|2485|chr1|−1|176136547|−0|r13|T1
QLEAQLEQIQK q value: 0.0027002 Tr_novel:TRUE RefSeq_Novel:FALSE
Search result spec prec mz: 665.3681 Actual spec prec mz: 665.36811
Fragments matched per AA: 2.36 Proportion of top 20 peaks matched: 0.35
Intensity
4e+06
2e+06
0e+00
y1
b1
b2*
b2
y2*
y2
b3
y3
b6*
b3*
y6
b4*
b4
y4*
y4
b9
b10*
y5
y5*
y5
b6
y6
y6*
y6
b7*
y9*
300
600
900
M/Z
B
Scatterplot of predicted elution time
Fitting R2: 0.86
Novel peptide residual Z score: 2.89
Number of peptides: 1955
Pred. hydrophobicity
60
40
20
0
0
20000
40000
60000
Experimental scan number
C
Distributions of residuals from best−fit line
of predicted RT vs Expt. scan number
Line: Z score of novel peptide
Z: 2.89
count
600
400
200
0
−10
−5
0
5
10
Z scores of residuals from best linear fit
